# Loss of endothelial *ZEB1* enhances neovascularisation during choroidal neovascularisation

**DOI:** 10.1038/s42003-026-10630-0

**Published:** 2026-07-07

**Authors:** Nicholas Beazley-Long, Joseph L. Horder, Kathryn R. Green, Joshua H. Bourne, Amy P. Lynch, Nada S. Ahmed, Zarah B. Tabrizi, Guillermo Diez-Pinel, Christopher P. Carroll, Claire L. Allen, Charles T. Cresswell, Poppy E. Harris, Isabela Ferreira, Diogo Mosqueira, Julie Rayes, Nigel P. Mongan, John King, Chris Denning, Alan McIntyre, Marc P. Stemmler, Simone Brabletz, Thomas Brabletz, David O. Bates, Andrew V. Benest

**Affiliations:** 1 Endothelial Quiescence Group COMPARE and Centre for Cancer Sciences School of Medicine, Biodiscovery Institute, University of Nottingham, Nottingham, UK; 2 Data Integrity and QA Team, Faculty of Health and Life Sciences, University of Bristol, Bristol, UK; 3 Institute of Cardiovascular Sciences, University of Birmingham, Edgbaston, Birmingham, UK; 4 Hypoxia and Acidosis Group Centre for Cancer Sciences, School of Medicine, BioDiscovery Institute, University of Nottingham, Nottingham, UK; 5 Tumour Vascular Biology Group COMPARE and Centre for Cancer Sciences School of Medicine, Biodiscovery Institute, University of Nottingham, Nottingham, UK; 6 Stem Cell Biology Group COMPARE and Centre for Cancer Sciences, School of Medicine, Biodiscovery Institute, University of Nottingham, Nottingham, UK; 7 Biodiscovery Institute, School of Veterinary Medicine and Science, University of Nottingham, Nottingham, UK; 8 Department of Pharmacology, Weill Cornell Medicine, New York, NY USA; 9 School of Mathematical Sciences, University of Nottingham, Nottingham, UK; 10 Friederich-Alexander-Univerisity Erlangen, Department of Experimental Medicine 1, Nikolaus-Fiebiger Center for Molecular Medicine, Erlangen, Germany; 11 Present Address: Centre for Inflammatory Diseases, Department of Medicine, School of Clinical Sciences, Monash Medical Centre, Monash University, Clayton, VIC Australia

**Keywords:** Angiogenesis, Gene regulatory networks

## Abstract

Choroidal neovascularisation (CNV) is a hallmark of wet/neovascular age-related macular degeneration (wAMD), characterized by aberrant blood vessel growth from the choroid into the retina. Both pathological angiogenesis and inflammation contribute to disease progression. Here we show that analsysis of single-cell RNA sequencing of experimental CNV lesions revealed upregulation of *Zeb1* in angiogenic endothelial cells (ECs). We generated an endothelial-specific *Zeb1* knockout (*Zeb1*^iECKO^) mouse model to assess its functional role. CNV was induced via laser photocoagulation, and vascular leakage and inflammation were evaluated using fluorescein angiography, immunohistochemistry, and transcriptomic analyses. *Zeb1*^iECKO^ mice exhibited increased fluorescein leakage and enhanced vascular invasion during CNV, indicating destabilized neovascular structures. However, leukocyte infiltration within CNV lesions was not elevated. In vitro, *ZEB1* knockdown in human ECs led to downregulation of inflammatory signalling pathways and reduced expression of adhesion molecules in response to TNF-α stimulation yet retained angiogenic capacity. ZEB1 coordinates angiogenic and inflammatory responses in CNV. Its loss enhances neovascularisation without promoting inflammation, suggesting a potential therapeutic target for modulating pathological angiogenesis in wAMD while minimizing inflammatory damage.

## Abstract (web summary)

Loss of endothelial ZEB1 enhances pathological choroidal neovascularisation without increasing inflammatory activation, revealing a context-dependent role for ZEB1 in regulating endothelial angiogenic and metabolic responses.

## Introduction

Wet age-related macular degeneration (wAMD) is a major cause of vision loss in individuals aged over 60, characterised by the growth of abnormal blood vessels from the choroid into the retina via a process known as choroidal neovascularisation (CNV). Newly formed vessels are fragile, prone to leakage and haemorrhage, leading to retinal damage, macular oedema, and permanent central vision loss. The pathogenesis of wAMD involves complex interactions between genetic susceptibility, oxidative stress, inflammation, and angiogenesis^1^. Vascular endothelial growth factor-A (VEGF-A) is the primary driver of angiogenesis in wAMD, stimulating endothelial cell (EC) proliferation and increasing permeability, which contribute to CNV^2,3^. Current treatments for wAMD focus primarily on inhibiting VEGF-A activity. Anti-VEGF-A therapies, delivered by intravitreal injections, include ranibizumab, bevacizumab, and aflibercept, and have revolutionised the management of wAMD^4^. Clinical trials have demonstrated that anti-VEGF-A therapy stabilises vision in the majority of patients and can even improve visual acuity in some cases^5^. Despite the success of anti-VEGF-A therapy, a significant subset of patients experience an incomplete response or develop resistance to treatment^6^, requiring frequent injections^7^. To address these challenges, dual-target agents such as faricimab, which target both VEGF-A and angiopoietin-2 (Ang2), have shown promise in extending the duration of treatment efficacy and improving clinical outcomes^8^. Furthermore, ongoing research is investigating alternative pathways such as the Wnt/β-catenin signalling cascade, fibroblast growth factors (FGFs), alternative splicing^9^, extracellular components^10^ and complement inhibition as potential therapeutic targets^11^. Such therapeutic developments signal a shift toward multi-targeted therapies in wAMD, which may offer more durable and personalised treatment options. However, angiogenic behaviour requires the complex interplay of a multitude of cytokines -not just VEGF-A- and EC phenotypic switching. Therefore, directly targeting endothelial cells and leveraging their inherent plasticity, rather than targeting specific cytokines^12^ may provide a more effective therapeutic strategy^13^.

Angiogenesis is the formation of new blood vessels from an existing vasculature and is regulated through dynamic changes in EC behaviour and their interactions with the surrounding tissue. This complex process leads to the differentiation of quiescent ECs into several potential angiogenic phenotypes^14^; including migratory tip cells^15^ and proliferative stalk cells^16^. Additional EC sub-populations with different phenotypes have been identified by transcriptomic analysis^17,18^ but the functional relevance remains to be explored. Tip and stalk cells are transient identities required to increase vascularity of a given tissue^19^. Once tip cells have anastomosed, their plastic phenotype reverts to a functional quiescent state. Transcriptomic techniques, including microarray analysis and, more recently, single-cell RNA sequencing (scRNA-seq) of angiogenic ECs, have significantly advanced our understanding of gene expression dynamics during angiogenesis^20,21^. While earlier methods provided valuable insights into population-level gene profiles, single-cell sequencing has revealed EC subpopulation heterogeneity and uncovered distinct transcriptional programs that drive different stages and modes of angiogenic behaviour. ECs exhibit significant diversity, with differences in transcriptomics, proteomics, morphology, and function at various levels, ranging from tissues and organs to interorgan and arterial, capillary, or venous localization^18,22,23^.

Using an unbiased approach to sequence RNA from single ECs during choroidal neovascularisation identified zinc-finger E-box binding protein 1 (ZEB1, formally known as TCF8) as significantly upregulated in angiogenic endothelial cells^13,17^ a upregulation not specifically annotated nor explored. Human *ZEB1* is a 1117 amino acid transcription factor comprising two C2H2-type zinc finger clusters positioned at the N- and C-termini, separated by a central non-DNA-binding homeodomain. It is highly conserved between human (*ZEB1*) and murine (*Zeb1*) forms (https://www.ensembl.org/Homo_sapiens/Gene/Compara_Ortholog?g=ENSG00000148516;r=10:31318495-31529814). Such a structure allows interactions with E-box or E-box-like motifs in target promoters and transcriptional co-regulators^24^. In vivo studies using endothelial-specific *Zeb1* knockout (KO) models have revealed that *ZEB1* is required for tumour angiogenesis: EC-specific *ZEB1* deletion impairs tumour angiogenesis and drives vascular normalisation, largely through repression of TGF-β signalling^25^. However, studies using global *Zeb1* KO mice (TCF8-null mice) demonstrated an inconsistent phenotype: enhanced tumour angiogenesis and growth compared to controls^26^. EC-specific *Zeb1* KO reduced bone vascularisation and formation via DLL4/Notch signalling^27^. During retinal vascular development, endothelial *ZEB1* expression correlates with vascular quiescence entry, and its deletion disrupts vessel stabilisation through dysregulation of the Wnt/β-catenin-FOXO1 axis^28^. *Zeb1* has also been implicated in corneal endothelial cell plasticity. Loss-of-function studies using CRISPR-Cas9 in corneal endothelial cells induced an epithelial-like phenotype reminiscent of Posterior Polymorphous Corneal Dystrophy, with associated apoptosis and barrier dysfunction^29^.

The functional implications of *Zeb1* expression in CNV pathophysiology remain unknown, prompting us to investigate its role in endothelial plasticity and inflammation. Our working hypothesis was that endothelial ZEB1 would be required for stabilising neovascular structures during choroidal neovascularisation and its loss would therefore affect pathological angiogenesis through actions on endothelial plasticity, vessel destabilisation and inflammation.

## Results

### ZEB1 is enriched in angiogenic endothelial cells during choroidal neovascularisation

**Fig. 1:**
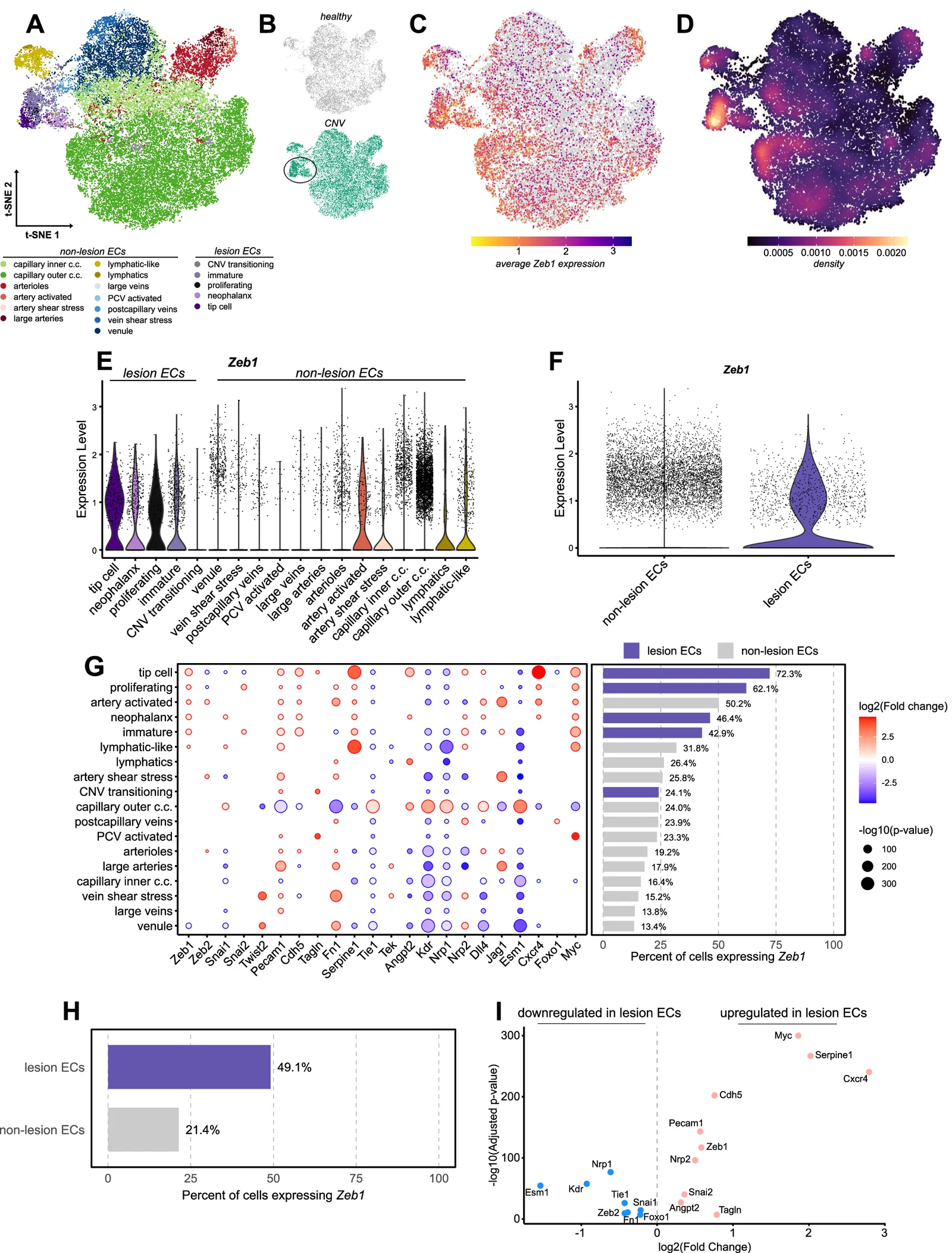
Reanalysis of a publicly available choroidal neovascularization (CNV) endothelial cell (EC) scRNA-seq dataset identified an increase in proportion of cells expressing Zeb1 in lesion ECs compared to non-lesion ECs. **A** t-SNE representation of the data with EC phenotype populations highlighted. The t-SNE dimensions and cluster annotations were extracted from the original publication of the dataset. **B** t-SNE from (**A**) split by healthy (top) and CNV (bottom) conditions. A population of cells only present in the CNV condition are circled and are deemed the lesion population of ECs. **C** Expression of Zeb1 mapped to (**A**). While Zeb1 appeared to be expressed in most EC populations, there was a increase the number of cells expressing Zeb1 in the lesion ECs (circled). **D** Density plot of Zeb1 expression. An increased density of Zeb1 expression was observed in the lesion population of ECs (circled), corroborating (**C**). **E** Violin plot of Zeb1 expression across all EC phenotypes. Zeb1 is expressed in greater proportion of lesion EC phenotypes compared to non-lesion, particularly tip and proliferating ECs. While the outer capillary EC population shows a large number of Zeb1 expressing cells, this makes up a reduced proportion of the phenotype compared to lesion EC phenotypes, as seen in (**G**). **F** Violin plot of Zeb1 expression in EC phenotypes grouped into non-lesion and lesion ECs. Lesion ECs show an increased proportion of cells expressing Zeb1 compared to non-lesion. **G** Dotplot demonstrating the differential expression of epithelial-mesenchymal-transition (EndoMT) drivers and canonical EC markers across EC phenotypes and the proportion of cells in the phenotype that express Zeb1. Zeb1 is upregulated in lesion ECs and shows the greatest proportion of expression in tip and proliferating ECs. It is important to note that the nature of the differential expression analysis considers cells that do not express Zeb1. **H** Proportion of grouped lesion and non-lesion EC phenotypes that express Zeb1. Zeb1 is expressed in greater proportion in lesion ECs. **I** Volcano plot of differential expression of genes in (**G**) across grouped lesion and non-lesion EC phenotypes.

During CNV, invading ECs acquire an angiogenic phenotype, providing the plasticity to proliferate and migrate into the retinal layers^18^. The canonical model of sprouting angiogenesis describes this process as a reversible display of transient EC phenotypes such as tip, stalk and phalanx cells^18^. A modification to this model proposes the loss of quiescence and increased motility are analogous to epithelial-to-mesenchymal transition (EMT), and therefore to endothelial-to-mesenchymal transition (endoMT)^30^. However, angiogenesis is a reversible process and thus represents only a partial transition. To determine whether such phenotypic switching is transcriptomically evident during CNV, we first asked whether an EndoMT-like phenotype^30^ was present within angiogenic EC populations in a publicly available scRNA-seq dataset of murine ECs isolated from CNV tissue. We probed a scRNA-seq dataset^18^ that consisted of 28,337 choroidal ECs and 13,769 genes (see Suppl. Data 1). Using the t-SNE embedding and cell annotations from the original publication^18^, we visualised the distinct EC subpopulations representing arterial, capillary, venous, lymphatic and angiogenic phenotypes (Fig. 1A). When stratified by condition, a discrete cluster of ECs was present exclusively in CNV cells compared to the healthy population and was defined as the lesion EC population (Fig. 1B), as previously described in the original publication^18^. Mapping *Zeb1* expression onto the t-SNE revealed that *Zeb1* was broadly expressed across EC phenotypes, with a notable enrichment within lesion ECs (Fig. 1C). A density plot of *Zeb1* expression further confirmed this increase in the lesion population (Fig. 1D). *Zeb1* expression was found to be most highly expressed in tip and proliferating ECs, cell states enriched in the lesion cluster, while expression in non-lesion ECs was comparatively lower (Fig. 1E, F).

We next assessed the differential expression of *Zeb1*, EndoMT-related and canonical endothelial genes across EC phenotypes (Fig. 1G). *Zeb1* was found to be upregulated in tip, proliferating, neophalanx and immature ECs of the lesion (Fig. 1G), indicating an induction of *Zeb1* expression in response to laser-induced injury. Furthermore, *Zeb1* was also upregulated in the activated artery phenotype (Fig. 1G), suggesting *Zeb1* is more prevalent in active EC phenotypes. Canonical EC markers *Pecam1* and *Cdh5* and mesenchymal marker *Serpine1* were also upregulated in lesion EC phenotypes (Fig. 1G), reflecting a potential hybrid endothelial/mesenchymal state of ECs in the lesion. Notably, *Serpine1* showed upregulation in tip cells (Fig. 1G), which suggests this population may exhibit a more mesenchymal-like phenotype compared to other choroidal EC clusters. Quantification of *Zeb1*-positive cells revealed that tip and proliferating ECs in the lesion had the highest proportion of *Zeb1* expressing cells, at 72.3% and 62.1%, respectively (Fig. 1G). Furthermore, grouping of EC phenotypes into lesion and non-lesion categories (Fig. 1H) showed 49.1% of lesion ECs expressed *Zeb1* compared to 21.4 of non-lesion ECs. This is highly suggestive that *Zeb1* plays a crucial role in the endothelial response to CNV. Differential expression analysis of the genes in Fig. 1G grouped by lesion and non-lesion status (Fig. 1I) reflected the results from Fig. 1G, with an upregulation of *Zeb1*, *Pecam1*, *Cdh5* and *Serpine1* in lesion ECs. Collectively, these findings indicate that *Zeb1* is upregulated in ECs within CNV lesions, particularly in angiogenic and proliferative phenotypes, suggesting a role for *Zeb1* in driving endothelial plasticity during pathological neovascularisation.

### In silico deletion of ZEB1 predicts inflammatory and angiogenic consequences

**Fig. 2:**
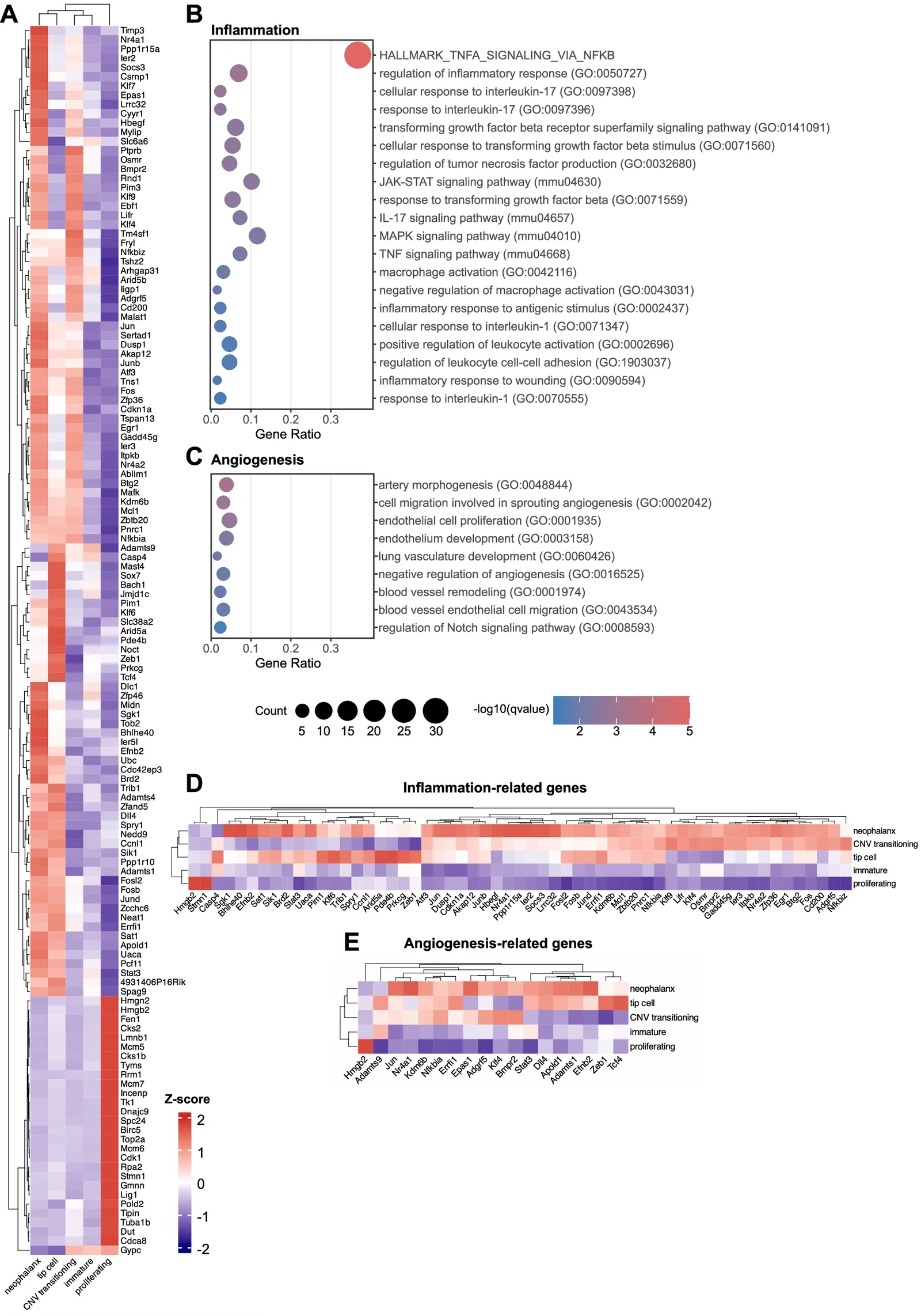
In-silico KO analysis of Zeb1 in lesion ECs predicted a perturbation of genes related to inflammation and angiogenesis. **A** Heatmap of the 133 genes predicted to be differentially regulated (DR) upon in-silico Zeb1 KO in lesion ECs. A cluster of genes that showed increased expression in tip cells compared to other lesion EC phenotypes is highlighted and contains Zeb1. A distinct cluster of genes upregulated in proliferating ECs was also identified in the Zeb1 DR genes. **B**, **C** Functional enrichment analysis of the 133 genes predicted to be DR upon in-silico Zeb1 KO in lesion ECs identified processes and pathways related to inflammation (**B**) and angiogenesis (**C**). **D** Heatmap of the inflammation-related DR genes identified in (**B**). Neophalanx, CNV transitioning and tip cell lesion EC phenotypes displayed an increased inflammatory signature compared to immature and proliferating ECs. A cluster consisting of four genes (highlighted); Zeb1, Prkcg, Pde4b, and Arid5a displayed increased expression in tip cells compared to other lesion EC phenotypes. **E** Heatmap of the angiogenesis-related DR genes identified in (**C**). Neophalanx, tip cell and CNV transitioning phenotypes showed an increased expression of angiogenesis-related genes compared to immature and proliferating EC phenotypes. 2 genes had a greater expression in tip ECs compared to other lesion EC phenotypes; Zeb1 and Tcf4.

In-silico deletion of *Zeb1* in lesion-associated endothelial cells (ECs) revealed coordinated dysregulation of inflammatory and angiogenic gene programmes. The differential expression analysis identified 133 predicted *Zeb1*-responsive genes (Fig. 2A), including a cluster enriched in tip-cell phenotypes that exhibited increased expression relative to other lesion EC states and contained *Zeb1*. Functional enrichment analyses (Fig. 2B, C) demonstrated strong over-representation of inflammatory pathways, including TNFα/NF-κB, IL-17, MAPK and JAK–STAT signalling, in addition to angiogenesis-related processes such as artery morphogenesis, endothelial proliferation and vessel sprouting. When mapped back to EC phenotypes, the inflammation-related gene set (Fig. 1G) showed that neophalanx, tip and CNV-transitioning ECs exhibited a heightened inflammatory signature compared with immature and proliferating ECs, with four genes (*Zeb1*, *Prkcg*, *Pde4b* and *Arid5a*) forming a distinct inflammatory module enriched within tip cells (Suppl. data 2). Similarly, the angiogenesis-related genes (Fig. 2E) were preferentially upregulated in neophalanx, tip and transitioning ECs, while *Zeb1* and *Tcf4* displayed notably higher expression specifically in tip ECs. Together, these data (Fig. 2A–E) predict that *Zeb1* loss perturbs both inflammatory and angiogenic programmes in lesion ECs.

### Conditional knockout of ZEB1 in adult male mice induces greater neovascularisation during CNV

**Fig. 3:**
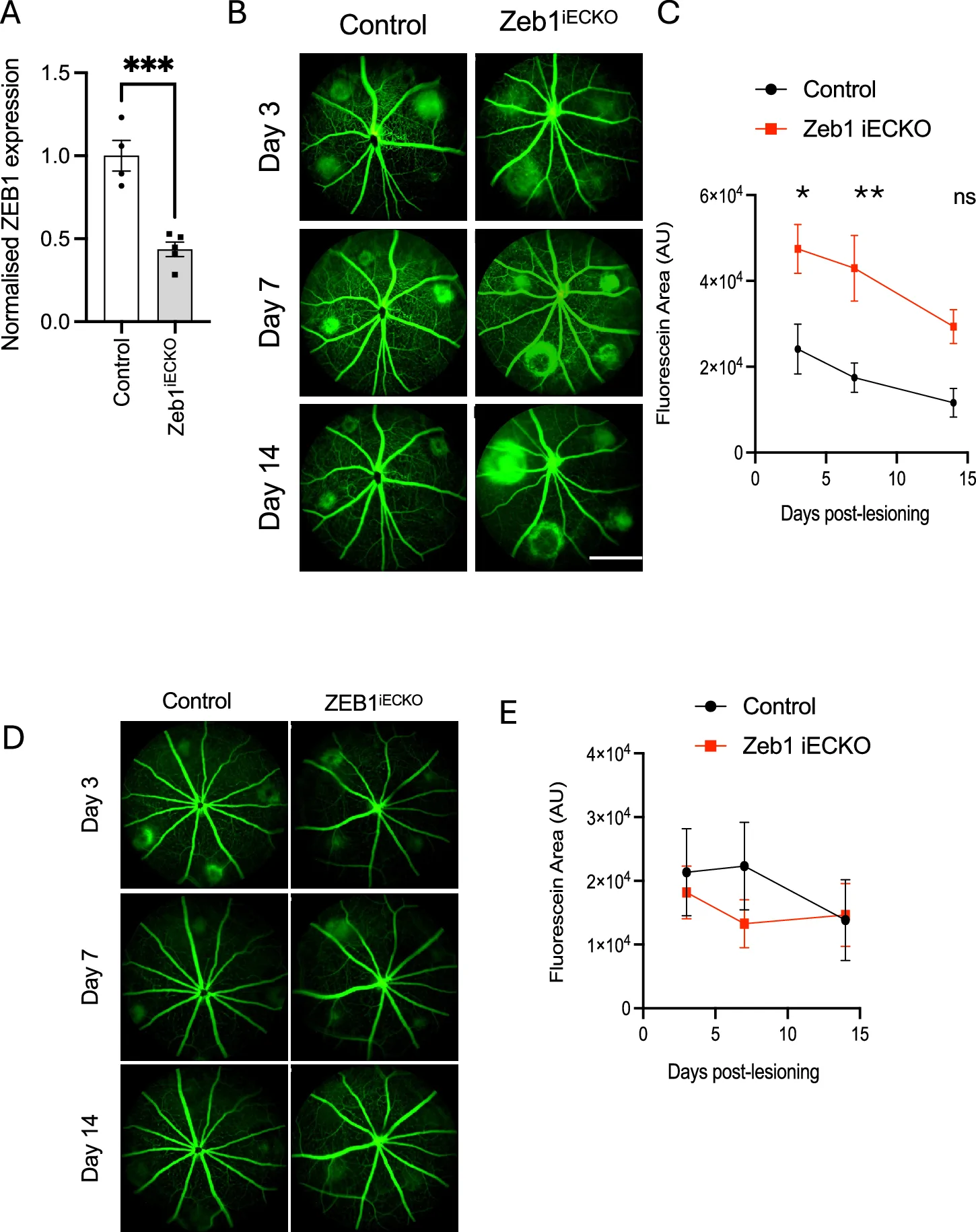
Zeb1^iECKO^ enhances fluorescein leakage in males in vivo. **A** Droplet digital PCR analysis of *Zeb1* expression in lung endothelial cells (ECs) isolated from control mice (white,-filled circles, *n* = 4) and Zeb1^iECKO^ mice (grey-filled squares, *n* = 5) confirms significant endothelial-specific knockout of Zeb1 (*p* = 0.006, unpaired t-test). **B** Fundus fluorescein angiography (FFA) was performed at days 3, 7, and 14 following laser-induced choroidal neovascularization (CNV). **C** Male Zeb1^iECKO^mice (*n* = 9) exhibited significantly increased fluorescein leakage (arbitrary units, AU) compared with male controls (*n* = 7) at day 3 (*p*adj = 0.027) and day 7 (*p*adj = 0.0061), with a non-significant increase at day 14 (*p*adj = 0.1049). **D**, **E** ZEB1^iECKO^ had no impact on fluorescein fundus angiography in female mice. Statistical analysis was performed using two-way ANOVA with Tukey’s multiple comparisons test. Data are presented as mean ± SEM. Abbrev. iECKO: inducible endothelial cell knock out. Black line = control mice. Red line = ZEB1^iECKO^.

To determine whether *Zeb1* expression correlates with in increased angiogenic activity, we generated an EC-specific, inducible knockout mouse in which *Zeb1* was deleted specifically in VE-Cadherin expressing cells. Adult mice were treated with tamoxifen to induce *cre* recombination, and CD31^+^ ECs were isolated from lungs at the end of the experimental procedures. Normalised *Zeb1* mRNA was reduced by greater than 50% (Fig. 3A, *p* = 0.0006, *n* = 4 control, *n* = 5 KO) in adult mice, demonstrating gene knockout at a level consistent with other adult EC-specific knockout models. CNV was induced, and fundus fluorescein angiography (FFA) was performed. At each time point post CNV, Zeb1^iECKO^ (*n* = 9) mean lesion area was greater than their littermate controls (*n* = 5), at days 3 (*p* = 0.0275), 7 (*p* = 0.0061) and 14 (*p* = 0.1049) (Fig. 3B, C). This effect was observed in male mice, but not in female mice (Fig. 3D, E).

**Fig. 4:**
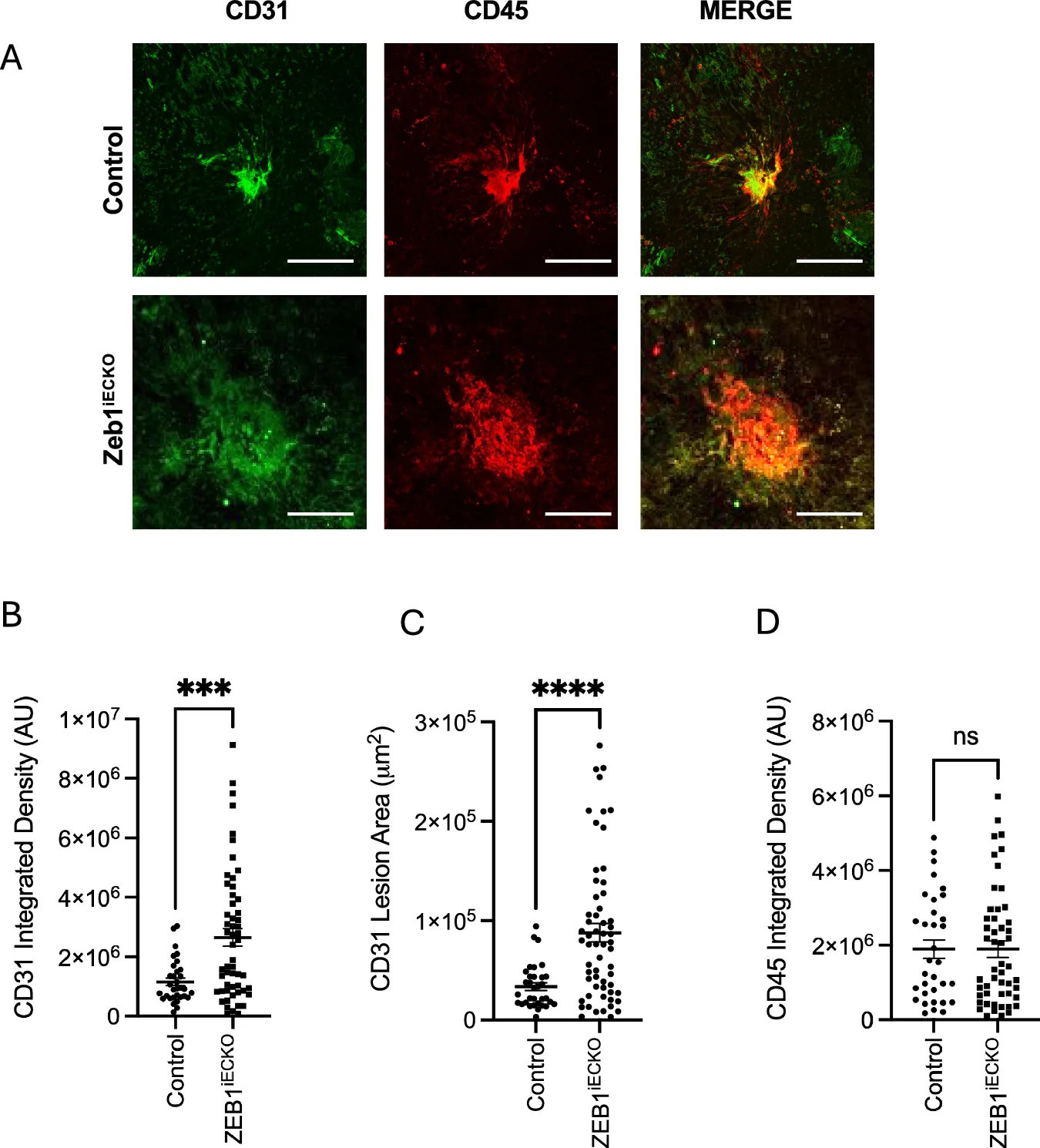
Zeb1^iECKO^ enhances choroidal neovacularisation in vivo. **A** Excision and staining of lesioned choroids followed by immunostaining for CD31 and CD45. **B** CD31 intensity and area was significantly increased following Zeb1^iECKO^, *p* < 0.004) and significantly greater (**C**) CD31^+^ area, (*p* < 0.0001). **D** Leukocyte accumulation (CD45^+^ cell infiltrate) was not altered (**C**, *p* = 0.9896). Data are presented as mean ± SEM and analysed by unpaired t-test. Abbrev. iECKO: inducible endothelial cell knock out. AU Arbitrary Units.

The sustained and greater lesion area in *Zeb1*^iECKO^ mice could be due to either increased vessel leakage alone or with increased lesion vascularity. Upon completion of the study, eyes were dissected and choroids were prepared for immunohistochemistry and confocal microscopy. The CD31^+^ vascular invasion in the lesion was significantly enhanced in *Zeb1*^iECKO^ mice as demonstrated by CD31^+^ intensity (Fig. 4A, B, *p* = 0.004) and CD31^+^ area (Fig. 4A, C, *p* < 0.0001). Therefore, *Zeb1*^iECKO^ resulted in increased vascularity. In contrast to the observed angiogenic response, CD45^+^ leukocyte invasion was still evident (and qualitatively distinct) in the control but was not quantitatively altered by *Zeb1*^iECKO^ (Fig. 4A, D, *p* = 0.09986). Thus, the increased angiogenic remodelling observed following *Zeb1* knockout occurred independently of any increase in CD45^+^ leukocyte-mediated inflammation.

### *ZEB1* knockdown in HUVECs alters inflammatory and angiogenic transcriptional programs and validates in-silico modelling

**Fig. 5:**
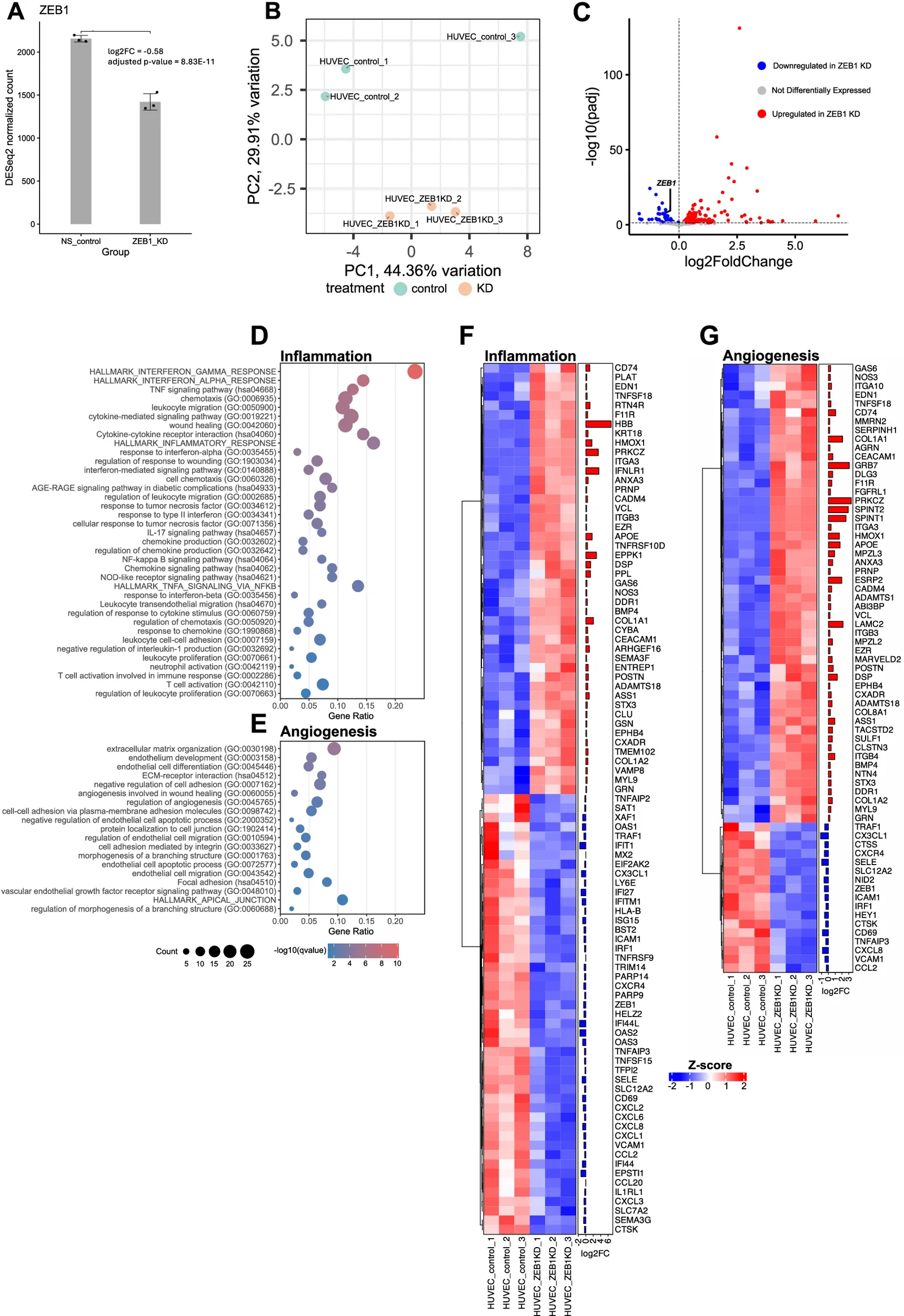
Bulk RNA-seq analysis of ZEB1 knockdown (KD) in human umbilical vein endothelial cells (HUVECs) resulted in 217 significant differentially expressed genes (DEGs) and regulation of inflammation and angiogenesis-related processes. **A** Mean DESeq2 normalized counts of ZEB1 in non-silenced (NS_control) and ZEB1 KD (ZEB1_KD) (*n* = 3 per group) revealed significant KD of ZEB1 (Benjamini-Hochberg adjusted *p*-value determined by DESeq2 = 8.83E-11 and log2FC = −0.58). Group means ± standard deviation and individual sample values are shown. **B** PCA analysis identified ZEB1 KD to be the second highest source of variation in the RNA-seq data. **C** Volcano plot of the 217 DEGs upon ZEB1 KD. ZEB1 is labelled. **D**, **E** Functional enrichment analysis of the 217 DEGs identified processes and pathways related to inflammation (**D**) and angiogenesis (**E**). **F** Heatmap of the inflammation-related DEGs identified in (**D**). Log2FC of the DEGs is represented as a barplot. **G** Heatmap of the angiogenesis-related DEGs identified in (**E**). Log2FC of the DEGs is represented as a barplot.

To experimentally validate the regulatory role of *ZEB1* suggested by our in-silico analyses, we performed bulk RNA-sequencing in human umbilical vein endothelial cells (HUVECs) following siRNA-mediated *ZEB1* knockdown (KD). Differential expression analysis confirmed a significant downregulation of *ZEB1* expression (log2FC = −0.58, adjusted *p*-value = 8.83E-11) compared with non-silenced controls (Fig. 5A). Principal component analysis (PCA) revealed that *ZEB1* KD represented a major source of transcriptional variation, clearly separating KD and control samples (Fig. 5B). Differential expression analysis identified 217 significantly differentially expressed genes (DEGs) upon *ZEB1* KD (Fig. 5C). Functional enrichment analysis of these DEGs revealed enrichment of processes related to both inflammation and angiogenesis (Fig. 5D, E) consistent with the in-silico KO of *Zeb1* in the lesion ECs of the CNV scRNA-seq analysis. Inflammatory pathways included *TNF*, *NF-κB*, *IL-17* and interferon signalling, alongside regulation of leukocyte adhesion, chemotaxis and cytokine production, suggesting that *ZEB1* influences endothelial immune responsiveness. Angiogenesis-related terms encompassed endothelial migration, focal adhesion, vascular morphogenesis and extracellular matrix organisation, implicating *ZEB1* in the maintenance of endothelial structure and vessel remodelling. 93 of the 217 DEGs were related to inflammation (Fig. 5F, Suppl. data 2) and 69 were related to angiogenesis (Fig. 5G, Supplementary data 2). *ZEB1* KD regulated inflammation and angiogenesis-related genes in both up and down directions, suggesting *ZEB1* KD perturbs these signatures as a whole, rather than increasing or decreasing specifically. Together, these results demonstrate that *ZEB1* loss in HUVECs induces dysregulation of inflammatory and angiogenic programs, validating predictions from in-silico modelling and supporting a role for *ZEB1* as a central transcriptional regulator of endothelial activation and remodelling. Expansion of the associated genes into a heatmap (Fig. 5B) indicates enrichment of downregulated inflammation-related genes. Specifically, genes such as *CXCL10, IFIT1, OAS2, and SELE*, which are critical during inflammatory responses, exhibit significant downregulation in the *ZEB1* knockdown cells. This suggests that *ZEB1* plays a role in regulating the transcription of inflammatory pathways in endothelial cells.

### ZEB1 prevents normal inflammation but retains angiogenic activity

**Fig. 6:**
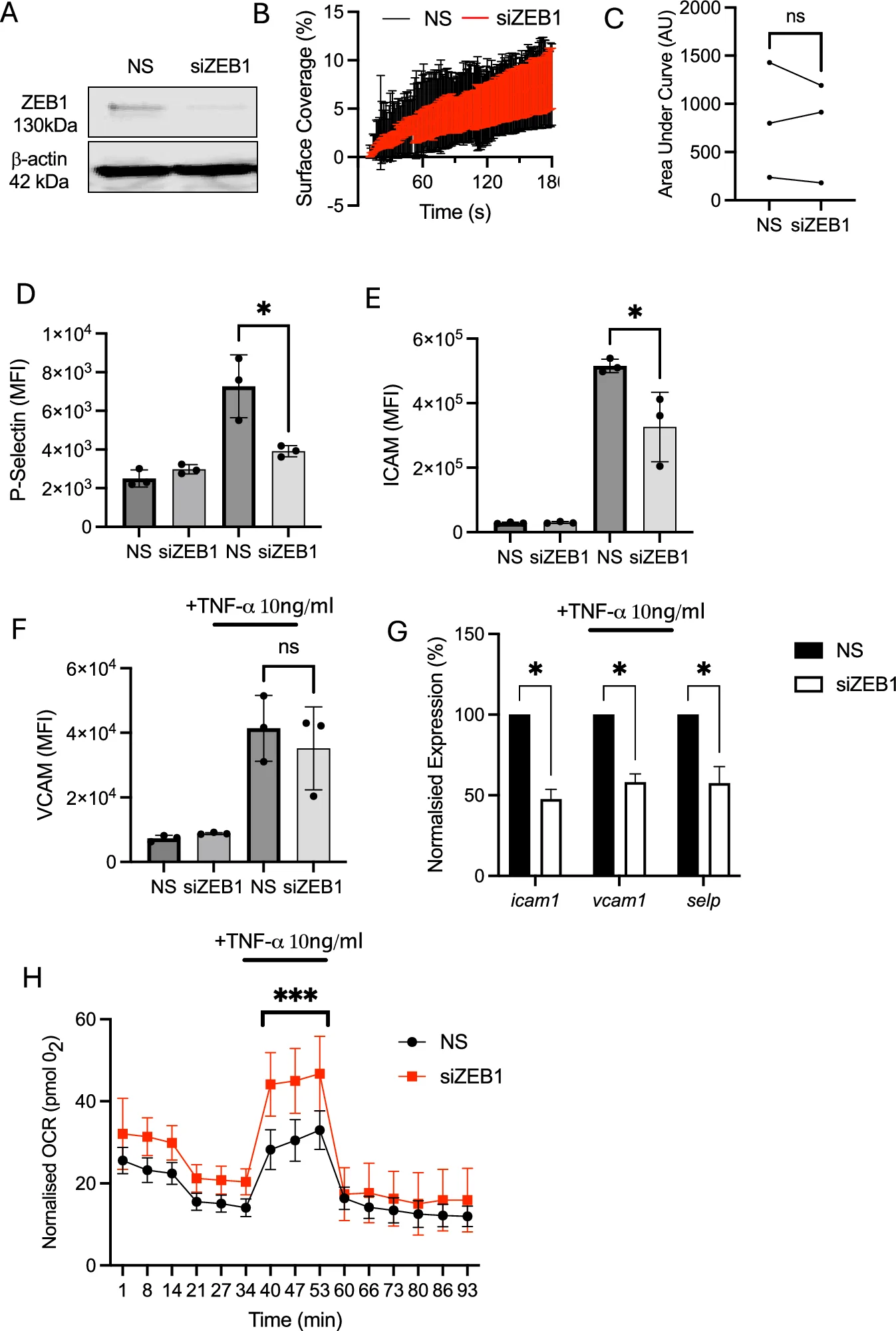
ZEB1 prevents normal inflammation but retains angiogenic activity. **A** Representative Western blot demonstrating efficient knockdown of human ZEB1 in HUVECs following transfection with siRNA targeting ZEB1 compared with non-silencing control (NS) siRNA. **B**, **C** ZEB1 knockdown did not alter adhesion of DiOC6-labelled whole blood cells to HUVEC monolayers under basal conditions (paired t-test, *p* = 0.6146, *n* = 3). However, ZEB1 knockdown (72 h) impaired the endothelial response to TNF stimulation, significantly reducing surface expression of P-selectin (**D**, *p* = 0.02) and ICAM1 (**E**, *p* = 0.04), while VCAM1 expression was unchanged (**F**, *p* = 0.5), quantified as median fluorescence intensity (MFI). Data are presented as mean ± SEM, *n* = 3. **G** qPCR analysis demonstrated reduced expression of ICAM1, VCAM1 and SELP following ZEB1 knockdown (*p* < 0.05, T-test). Seahorse metabolic flux analysis revealed increased endothelial metabolic activity following ZEB1 silencing, with a pronounced elevation in oxygen consumption rate (OCR) and maximal respiration following FCCP treatments, *n* = 6, mean ± SD, **p < 0.0002, two-way ANOVA). MFI median fluorescence intensity.

*ZEB1* knockdown (Fig. 6A) in HUVECs had no effect of platelet adhesion (Fig. 6B), with there being no discernible difference in the level of adherence between NS and *ZEB1* knockdown HUVECs (Fig. 6B). Individual responses from three human blood donors showed no difference in adhesion (Fig. 6C, *p* = 0.6146). Under unstimulated conditions, there was no difference in expression of P-selectin, ICAM-1 or VCAM-1 between *ZEB1* knockdown cells and control. 10 ng/ml TNFα stimulation significantly increased expression of the three adhesion molecules (ICAM-1 *p* = 0.0002, P-selectin *p* = 0.007, VCAM1 *p* = 0.0045 *ZEB1* knockdown significantly reduced the expression of both P-selectin (Fig. 6D, *p* = 0.02) and ICAM-1 (Fig. 6E, *p* = 0.04) under TNFα-stimulated conditions. VCAM-1 was not significantly reduced (Fig. 6F, *p* = 0.5492). To corroborate the RNA-seq results (Fig. 5), qPCR shows significantly reduced *ICAM1* (Fig. 6G, supplementary data 3, *p* = 0.0011), *VCAM1* (Fig. 6G, supplementary data 3*p* = 0.0012), and *SELP* expression (Fig. 6G, supplementary data 3, *p* = 0.0145). Indicating *ZEB1* downregulation results in a reduced capability of HUVECs to respond to inflammatory stimuli, which could be due to altered vessel integrity or a reduction in chemokine responsiveness. However, to confirm if *ZEB1* downregulation impacted the angiogenic phenotype we assessed the cellular bioenergetics using real-time measurements of oxygen consumption rate (OCR) and extracellular acidification rate (ECAR). Angiogenic ECs display increased metabolic activity^31,32^ in choroidal ECs. *ZEB1* knockdown ECs exhibited a metabolically activated phenotype, displaying significantly elevated oxygen consumption under basal conditions and following uncoupling of oxidative phosphorylation with FCCP (Fig. 6H, *p* < 0.0002, 2 way- ANOVA), indicating an increased maximal respiratory capacity.

## Discussion

Our findings establish Zeb1 as a marker of angiogenic ECs within the lesion, surpassing other markers that are more tip-cell specific (Fig. 1G). To probe the functional role of Zeb1, we generated an inducible, endothelial-specific *Zeb1* knockout mouse (*Zeb1*^iECKO^) (Fig. 3A). Tamoxifen-induced deletion resulted in ~50% reduction of *Zeb1* mRNA in isolated lung ECs, consistent with effective recombination (Fig. 3A). Following CNV induction, *Zeb1*^iECKO^ male mice exhibited significantly larger lesion areas compared to controls at days 3 and 7 post-injury, with a trend persisting to day 14 (Fig. 3B, C). Confocal imaging of choroidal flat-mounts identified that this lesion enlargement was driven by increased vascularity, as demonstrated by enhanced CD31^+^ immunofluorescence intensity and area (Fig. 4A–C). Strikingly, despite the exaggerated angiogenic response, CD45^+^ leukocyte infiltration was not increased in *Zeb1*^iECKO^ lesions (Fig. 4D), indicating an uncoupling of angiogenesis from inflammation. This dissociation is further supported by in vitro data: *ZEB1* knockdown in HUVECs led to downregulation of multiple inflammatory pathways including TNF, IL-17, chemokine, and NOD-like receptor signalling (Fig. 5A, B). Moreover, *ZEB1*-deficient human ECs (HUVECs) showed significantly reduced TNFα-induced expression of adhesion molecules ICAM-1 and P-selectin (Fig. 6D–F), critical mediators of leukocyte recruitment and transmigration, while VCAM-1 expression was unaffected. Platelet adhesion assays indicated that this was not due to generalized endothelial dysfunction (Fig. 6B, C) as *ZEB1* knockdown cells still responded to TNFα. These transcriptional and functional changes suggest that *ZEB1* orchestrates endothelial inflammatory activation without impairing basal endothelial functions or angiogenic capacity. In wet-AMD, pathological angiogenesis is closely intertwined with chronic inflammation^33^, which drives fibrosis and irreversible vision loss through scarring^34^. Persistent leukocyte infiltration promotes fibroblast activation and extracellular matrix deposition, culminating in subretinal fibrosis that current anti-VEGF-A therapies inadequately address^33^. By suppressing endothelial inflammation via *ZEB1* deletion, our findings indicate it may be possible to permit neovascular expansion while limiting the inflammatory cascade that precipitates scarring.

To explore whether ZEB1 may directly regulate endothelial inflammatory adhesion molecules, we interrogated a recently published ZEB1 ChIPSeq dataset generated in melanoma cells^35^. In this dataset, ZEB1 binding was detected at the ICAM1 promoter, with enrichment increased following TNFα stimulation. Additional peaks were observed at the promoters of ICAM3 and ICAM5 under TNFα-treated conditions, whereas no binding peaks were detected at the VCAM1 or SELP loci. Although derived from a melanoma model, these data support the possibility that ICAM-family genes represent transcriptional targets of ZEB1 during inflammatory stimulation, suggesting that ZEB1 may directly regulate ICAM1 expression. In our data ZEB1 knockdown significantly reduced ICAM1 expression in TNFα-stimulated HUVECs, alongside a reduction in P-selectin (SELP) at both transcript and protein levels and a reduction in VCAM1 transcript abundance detected by RNA-seq and qPCR. However, VCAM1 protein expression was not significantly altered, likely reflecting the distinct trafficking and storage mechanisms that regulate endothelial adhesion molecules^36^. For example, P-selectin is stored within Weibel–Palade bodies and rapidly mobilised to the endothelial surface during inflammatory stimulation, whereas ICAM1 and VCAM1 are primarily regulated through transcriptional induction and protein turnover^36^. Interestingly, ZEB1 binding was also detected at the NFKB2 promoter in melanoma cells^35^, consistent with reports linking ZEB1 to NFκB-mediated inflammatory signalling^37,38^. However, ZEB1 knockdown in HUVECs did not significantly alter expression of NFκB family members in our RNASeq dataset, suggesting that this interaction may become more evident under stronger inflammatory stimulation. Together, these findings support a role for ZEB1 in regulating endothelial inflammatory responsiveness through modulation of adhesion molecule expression during TNFα stimulation.

A recurring theme in the literature is that ZEB1 appears to have divergent effects on angiogenesis depending on biological context. In developmental systems, including the postnatal retinal vasculature, ZEB1 has been reported to support endothelial proliferation and vascular growth^28^. Similarly loss of endothelial ZEB1 activity is causative in bone formation^27^ and corneal dystrophies, whereby loss of ZEB1 inhibits normal EC proliferation and induces a shift towards a mesenchymal phenotype^29,39–41^. Consistently in a lung cancer model ZEB1 activity has been associated with pro-angiogenic endothelial behaviour^25^. In contrast, our findings in the laser-induced CNV model demonstrate that endothelial loss of Zeb1 enhances pathological neovascularisation (in male mice). We propose that these observations are not contradictory but instead reflect a context-dependent role for ZEB1 in regulating endothelial cell state (Table 1). Angiogenesis in developmental tissues, tumours, and inflammatory injury models such as CNV occurs within markedly different microenvironmental conditions that include distinct extracellular matrices, immune cell interactions, and cytokine profiles. Despite a potential disparity it is important to note that global heterozygous ZEB1 knockout mice display enhanced angiogenesis during tumour growth^26^ and yet during corneal angiogenesis (where there is growth into an avascular tissue using a pan-tissue ZEB1 deletion) there is a reduction in EC growth^42^, which reinforces context dependency of ZEB1 activity as the determinant of outcome, rather than EC ZEB1 acting identically in all tissues. ZEB1 is a transcriptional regulator capable of integrating multiple signaling inputs and has been implicated in processes including endothelial plasticity, inflammatory signaling, and cell cycle regulation. Our data therefore support a model in which ZEB1 does not simply promote or inhibit angiogenesis universally, but instead modulates endothelial transcriptional programs that determine how endothelial cells respond to specific physiological or pathological cues. Within the CNV setting, where vascular growth occurs in the context of tissue injury and inflammation, ZEB1 appears to act as a regulatory brake that limits excessive neovascularisation. These findings highlight the importance of considering endothelial transcription factors within the context of the tissue environment in which angiogenesis occurs.

**Table 1:** Context-dependent vascular phenotypes following global and endothelial-specific ZEB1 loss

KO Type	Model / Tissue Context	Experimental Setting	Vascular Phenotype upon ZEB1 Loss	Interpretation	References
Global heterozygous KO	Tumor	Tumor growth	Enhanced angiogenesis	ZEB1 restrains angiogenesis in tumors	^26^
Global / pan-tissue KO	Cornea (avascular tissue)	Corneal angiogenesis	Reduced EC growth / reduced angiogenesis	ZEB1 promotes endothelial growth in avascular environments	^70^
EC-specific KO	Bone	Developmental	Impaired bone formation (vascular defect)	ZEB1 required for endothelial function in osteogenesis	^27^
EC-specific KO	Cornea	Corneal dystrophies	Reduced EC proliferation + mesenchymal shift	ZEB1 maintains endothelial identity and proliferation	^29,67–69^
EC-specific KO	Lung cancer	Tumor angiogenesis	Reduced pro-angiogenic endothelial behavior	ZEB1 promotes angiogenic EC phenotype in tumors	^25^
EC-specific KO	Retina (CNV model)	Laser-induced inflammatory neovascularization	Enhanced pathological neovascularization (male mice)	ZEB1 acts as a brake on angiogenesis in inflammatory injury	(this study)

*ZEB1* zinc finger E-box-binding homeobox 1, *KO* knockout, *EC* endothelial cell, *CNV* choroidal neovascularization.

There is published evidence suggesting that age-related macular degeneration (AMD) progression may be more pronounced in female patients^43^, yet murine models of choroidal neovascularisation (CNV) have reported sex dependent differences in neovascularisation, although the direction of this effect is not consistent across studies^44–46^. In our model, loss of endothelial ZEB1 did not appear to exert the same impact on CNV in females as observed in males, suggesting that the angiogenic consequences of endothelial ZEB1 loss may be sex dependent rather than reflecting an inherent difference in baseline CNV progression between the sexes. To the best of our knowledge, this is the first report describing a sex specific angiogenic phenotype associated with endothelial ZEB1 in CNV. This observation is notable because ZEB1 expression and activity are known to be regulated by sex hormone signalling pathways^47,48^, raising the possibility that hormone-dependent regulation of endothelial ZEB1 may contribute to sexually dimorphic angiogenic remodelling in pathological settings.

Our in silico knockout^49^ analysis further predicted disruption of TCF4, a mediator of canonical Wnt signalling. This observation is of particular interest because ZEB1 has previously been linked to Wnt-dependent mechanisms that promote the restoration of endothelial quiescence during developmental angiogenesis^28^. More broadly, this relationship may be relevant to endothelial disease heterogeneity. For example, Fuchs endothelial corneal dystrophy occurs more frequently in females than in males but is not uniformly associated with identical Wnt-related mechanisms, suggesting that additional disease-modifying factors may contribute to its pathogenesis in females^50^. In contrast, other corneal endothelial dystrophies, such as posterior polymorphous corneal dystrophy (PPCD), show no clear sex bias and are mechanistically linked to altered endothelial ZEB1 signalling^29^. Together, these observations raise the possibility that sex dependent modifiers of ZEB1-associated endothelial biology may influence vascular remodelling responses in different disease contexts.

Unravelling stromal remodelling as a consequence of anti-angiogenic therapy represents a potential therapeutic paradigm shift: instead of broadly inhibiting angiogenesis (as with VEGF-A blockade), selectively targeting endothelial inflammatory pathways via *ZEB1* modulation could promote more organised, inflammation-silent vascular regrowth, potentially improving tissue repair and visual outcomes. This hypothesis warrants further investigation into long-term fibrotic remodelling, including evaluation of matrix deposition and pericyte coverage in *Zeb1*^iECKO^ lesions. Current treatments for wet-AMD, primarily anti-VEGF-A therapies, effectively reduce pathological angiogenesis but often fail to fully prevent chronic inflammation and subsequent fibrotic scarring, which are major contributors to long-term vision loss. Our novel findings strongly indicate that targeting *ZEB1* could overcome these clinical limitations by uncoupling angiogenesis from inflammation, allowing for controlled vascular repair without triggering excessive leukocyte recruitment or fibrosis. Modulating *ZEB1* activity in endothelial cells therefore represents a promising strategy to not only limit aberrant vessel growth but also to reduce inflammation-driven scarring, addressing a critical unmet need in AMD therapy.

## Material and Methods

### Generation of endothelial, inducible Zeb1 Knockout mice

We have complied with all relevant ethical regulations for animal use. All animal studies were reported in accordance with the ARRIVE 2.0 guidelines and were approved by the University of Nottingham Animal Welfare and Ethical Review Body and conducted under a UK Home Office project licence in accordance with the Animals (Scientific Procedures) Act 1986. *Zeb1*-floxed mice^51^ were crossed with heterozygous C57BL/6-Tg(Cdh5-cre/ERT2)1Rha mice^52^ to generate a mixed *Zeb1*^fl/fl^ cdh5 Cre-ERT2^+/^^−^ and *Zeb1*^fl/fl^ cdh5 Cre-ERT2^−/−^ *Zeb1* mice for studies. Mice were housed in individually ventilated cages under controlled temperature and humidity conditions with a 12 h light/dark cycle and free access to standard chow and water. Animals were group-housed where compatible, with nesting material and environmental enrichment provided routinely. Cages were maintained by trained animal care staff under specific pathogen-free or equivalent barrier conditions according to institutional husbandry procedures.

Animals were acclimatised before experimental procedures and were monitored by trained staff throughout the study. Genetically modified animals and littermate controls were housed under equivalent conditions to minimise environmental variation between experimental groups.

All litters were treated with tamoxifen to control for possible off target effects of tamoxifen, therefore Cre negative mice were used as control mice for the *Zeb1*^fl/fl^ cdh5 Cre-ERT2^+/−^ KO mice that were termed *Zeb1*^iECKO^. Both sexes were used. Genotyping was performed by Transnetyx using the following primers: *Zeb1*fl_fwd, 5′-CGTGATGGAGCCAGAATCTGACCCC-3′; *Zeb1*fl_rev, 5′-GCCCTGTCTTTCTCAGCAGTGTGG-3′; *Zeb1*del_rev, 5′-GCCATCTCACCAGCCCTTACTGTGC-3′ and CRE Fwd 5’-GCG GTC TGG CAG TAA AAA CTA TC-3’ and Rev 5’-GTG AAA CAG CAT TGC TGT CAC TT-3’.

### Tamoxifen induction of *Zeb1* knockout

100 mg Tamoxifen (Sigma) was added to 1 mL 100% ethanol (Thermo) and heated gently to 37 °C until fully dissolved before the addition of 9 mL freshly autoclaved sunflower oil in a class 2 safety cabinet^53^. Adult mice between 8 and 12 weeks received daily doses of 1 mg/25 g i.p. tamoxifen in sunflower oil for 5 consecutive days. Animals were weighed and welfare check performed. Following treatment mice were left undisturbed for one week prior to laser choroidal neovascularisation.

### Induction of anaesthesia for laser-induced choroidal neovascularisation

Injectable anaesthetics were made up in water (0.125 mL ketamine (Ketastet, Zoetis, 100 mg/ml stock) and 0.147 mL medetomidine (Sedastart, Animalcare Limited, 1 mg/mL stock) and 0.728 mL sterile water. 100 μL of anaesthetic cocktail was administered i.p per 25 g body weight. General anaesthesia was confirmed by the absence of hind paw and eyelid reflexes to touch. CNV was induced using Micron IV^TM^ ophthalmoscope (Phoenix Technology Group Inc.) and a Meridian Merilas Green laser photocoagulator (532 nm, class 3b). The laser power, time, and spot size were 450 mW, 130 ms and 50 μm. Eyes were prepared for CNV by topically applying 5% phenylephrine hydrochloride and 0.8% tropicamide to dilate the pupil. Viscotears were applied. A single eye was used per mouse. The mouse and ophthalmoscope were positioned to focus onto the retina. A guide laser was used to find a region of the retina clear of major vessels, and the laser fired. 4 lesions were made per eye. Data are presented per lesion, with multiple lesions analysed per eye across multiple mice in each experimental group. Pre and post lesion images were taken to confirm successful rupture of Bruch’s membrane as per standard parameters^33^. 200 μL Atipamezole (Sedastop, Animalcare Limited, 5 mg/mL) was diluted with 800 μL sterile saline and to produce 1 mg/mL and 200 μL administered by i.p injection. Animals were monitored for adverse effects following tamoxifen induction, anaesthesia, ocular imaging and laser injury. Expected transient effects included mild post-anaesthetic lethargy and temporary ocular irritation. Animals were monitored for unexpected adverse events including weight loss, reduced mobility, abnormal posture, ocular opacity, infection, excessive distress or failure to recover normally from anaesthesia. No unexpected adverse events were observed. Humane endpoints were established before the study. These included persistent weight loss exceeding the approved threshold, failure to eat or drink, severe or persistent ocular damage, signs of infection, abnormal behaviour, prolonged distress, or failure to recover after anaesthesia. Animals were monitored at least daily during routine husbandry and more frequently following procedures, according to the severity and timing of the intervention. Any animal reaching a humane endpoint would be humanely killed using an approved Schedule 1 or otherwise authorised method.

### Fundus fluorescein angiography

On days 3, 7 and 14 post-lasering the mice underwent Fundus Fluorescein Angiography (FFA) to observe lesion growth and leakage. The mouse eye was prepared as before and then received an IP injection of 200 μL 10% sodium fluorescein. Approximately 3 min after i.p. injection the FITC channel was captured using the Micron program. Lesion size was measured and analysed taking the lesions area and integrated intensity (therefore presented as Arbitary Unit) using FIJI (NIH, USA) Lesions were analysed if they satisfied published criteria^33^.

### Immunohistochemical staining of whole-mount choroids

On day 14 post-lasering, mice were culled via cervical dislocation and the lasered eyes removed and fixed in 4% PFA for 1 hour. Eyes were twice washed and dissected to remove the ocular muscles, nerve, iris, lens and retina to expose the choroidal layers. The tissue was ‘petalled’ to allow flat-mounting. Choroids were washed in PBS for 2 hours with rocking and blocked in 0.22μm filter sterilised 4% Bovine Serum Albumin (BSA) and 0.5% Triton-X in PBS for 2 h. Choroids were subsequently incubated for 48 h (4 °C with rocking) in primary antibodies to CD45 (goat anti mouse, AF114, R&D systems) and CD31 (rat anti mouse, 550274 BD Bioscience) diluted in 4% BSA and 0.5% Triton-X in PBS. Choroids were washed in 0.5% Triton-X in PBS for 4 hours (changing every 30 min) on rocking platform. Secondary antibodies Anti-Rat Alexa Fluor 488 (A-11006, Invitrogen) and Anti-Rabbit Alexa Fluor 555 (A-31572, Invitrogen) were diluted in 1:500 along with DAPI 1:1000 in 4% BSA and 0.5% Triton-X in PBS (48 h on a rocking platform overnight at 4 °C). Choroids were washed with 0.5% Triton in PBS changing solution every 30 min for 4 h before 2 washes in PBS and mounted onto glass slides with Fluromount G mounting medium (00-4958-02, Thermo Fisher) topped with a coverslip and sealed using clear nail varnish at room. Slides were then stored at 4 °C. Choroids were imaged using a Leica TCS SPE Confocal Microscope. An 8 × 8 tile scan of the choroid was acquired at 20× magnification using confocal microscopy. CNV lesions were identified based on CD31-positive vascular signal, which was used to define the lesion boundary, consistent with established approaches for visualising neovascular complexes in whole-mount choroid preparations. Regions of interest (ROIs) were manually delineated around the CD31⁺ lesion area in ImageJ (NIH, USA). The CD31-positive area was used as a measure of lesion size, while CD45 fluorescence intensity within the same ROI was quantified as a measure of inflammatory cell infiltration. All lesions within each eye (up to four per eye, therefore only one choroid per mouse) were analysed individually. Quantification was performed using a consistent analysis workflow across all samples, with identical acquisition settings and threshold parameters applied to all images. Fluorescence intensity values are reported in arbitrary units (AU) derived from integrated fluorescence intensity within each ROI.

### Confirmation of *Zeb1* knockout

Mice were culled by cervical dislocation and lungs removed. Lungs were chopped and minced in ECGM and a cell suspension was achieved using a lung dissociation kit (Macsbeads, Miltenyi, 130-095-927) at 37 °C for 2 h. The resultant cell suspension was then passed through a 70 μm and then 30 μm cell strainer. The resultant suspension was centrifuged at 1100 rpm and the pellet resuspended with 10 μL CD31 Macsbeads (Miltenyi) and 90 μL PEB sorting buffer per 10^7^ cells before being added to an end-to-end rotator for 30 minutes at 4 °C. The labelled cells were then centrifuged at 1100 rpm at 4 °C, resuspended in 500 μL PEB per 10^7^ cells ready for magnetic cell sorting. Miltenyi LS columns were used to purify cells into CD31- and CD31+ fractions. Fractionated cells were stored in Trizol and RNA was extracted with chloroform/isopropanol. 1 μg DNAase treated RNA was reverse transcribed using the Takara PrimeScript RT Master Mix kit (RR036A, Takara). Taqman probes to murine *ZEB1* (Mm00495564_m1, Thermo) and GAPDH (Mm99999915_g1, Thermo) were used for QX100 droplet digital PCR (Biorad) for gene expression quantification.

### Human umbilical vein culture

Primary HUVECS (Promocell, Heidelberg) were cultured (37 °C, 5% CO_2_), and were routinely passaged (maximum six passages per batch of primary cells) at approximately 80% confluency in endothelial cell growth medium (ECGM, Promocell, Heidelberg). A total of 3 х 10^5^ HUVECs were grown per 6-well plate.

### Flow cytometry immunofluorescent staining

Unstimulated (control) or 24h-TNF-α-activated (10 ng/mL in media, 300-01A-50UG, Peprotech) HUVECs were dissociated from culture using ice-cold PBS-EDTA (2 mM) and gentle tapping/scraping. Non-specific antigen binding was blocked with 10% BSA-PBS with anti-human CD16/32 (65057-1-IG500, Thermo Fisher, 1:100), for 20 minutes on ice. Cells were pelleted and resuspended in an antibody cocktail of mouse anti-human CD62P-PE (P-selectin, 555524, BD Pharmingen), CD54-FITC (ICAM-1, Thermo, BMS108FI) and CD106-PerCP-Cy5 (VCAM-1, 563525, BD Pharmingen) or isotype controls. Cells were again pelleted and resuspended in PBS for acquisition using an Accuri C6 flow cytometer in a volume of 200 mL PBS with 1% BSA and a minimum of 1 х 10^4^ cells acquired. Dead cells/debris was gated out. Data were analysed using FlowJo v10.

### Real time analysis of oxygen consumption and extracellular acidification rate

A total of 4 × 10^4^ HUVECs were plated onto a gelatinized XF96 plate (Agilent) in ECGM. Immediately prior to experimenting, media was changed to Seahorse XF media (per 100 mL: 97 ml Seahorse XF base media (102353-100 Agilent), 1 ml 100 mM Na pyruvate, 1 ml 200 mM L-glutamine, 1 mL 1 M glucose) and pH was corrected to pH 7.4. Cells were stimulated with 1.2 M oligomycin, 1 µM FCCP and 1 μM Rotenone.

### Leukocyte adhesion under flow

Pan-leukocyte adhesion was performed as previously described in refs. ^54,55^. In brief, venous blood was drawn from healthy, consenting volunteers into 3.2% citrated containers (BD) under ethical approval awarded by The University of Birmingham Research Ethics Committee (ref: UHSP/22/BTVR/07). Whole blood was perfused over TNF-α-activated (20 ng/mL, 24 h) HUVECs (Promocell), cultured on 2% gelatine, through a µ-Slide VI 0.4 (Ibidi, 80608). Blood was perfused at low arterial shear stress (10 dynes/cm^2^). Blood was treated with 2 µM DiOC6 (Thermo Fisher, D273) and 40 µM PPACK (Sigma, 520222) immediately prior to perfusion. Images were acquired simultaneously of two fields with Z-stacks using an EVOS M5000 Imaging system (Thermo Fisher), by fluorescence and transmitted light imaging (20х lens). Images were analysed using parameter specific semi-automated ImageJ scripts, as described in refs. ^56,57^.

### siRNA knockdown of *ZEB1*

HUVECs were cultured as described above and seeded at 2.5 × 10⁵ cells per well in 6-well plates in ECGM. After 24 h, the medium was replaced with 800 μL OptiMEM (Gibco, 31985062). For transfection, 30 μL of 20 μM siRNA SMARTpool (Dharmacon, Horizon Discovery) targeting ZEB1 or a non-silencing control was diluted in OptiMEM and combined with 15 μL Lipofectamine RNAiMAX (Thermo Fisher Scientific, 13778150) according to the manufacturer’s instructions. Cells were incubated with the transfection mixture for 4–6 h, after which the medium was replaced with fresh ECGM. RNA interference was performed using 20 nM ON-TARGETplus SMARTpool siRNA (targeting ZEB1(Gene ID: 6935; L-006564-01-0020; target sequences: CUGUAAGAGAGAAGCGGAA, CUGAAAUCCUCUCGAAUGA, GCGCAAUAACGUUACAAAU, GCAACAGGGAGAAUUAUUA). A non-targeting ON-TARGETplus control pool (D-001810-10-20; sequences: UGGUUUACAUGUCGACUAA, UGGUUUACAUGUUGUGUGA, UGGUUUACAUGUUUUCUGA, UGGUUUACAUGUUUUCCUA) was used as the control condition. Cells were incubated for 72 h post-transfection, after which ZEB1 knockdown was confirmed by Western blotting. RNA samples were extracted at 48 h post transduction^58^.

### Western blot analysis

Up to 40 μg total protein (estimated by Bradford reagent, Bio-Rad, #5000205) was loaded on to a gradient 14–20% miniProtean gel (Bio-Rad, #4561024) and electrophoresis performed using a Tris/glycine/SDS buffer (Bio-Rad, #1610732). Protein was blotted onto a nitrocellulose Trans-Blot Turbo membrane (Bio-Rad, #1704158) with two high MW transfer cycles. Membranes were blocked using 5 % BSA-TBS-0.1% Tween20 (TBST) for 1 h and 30 minutes, before incubating for 18 h on a roller at 4 °C in 1 % BSA-TBST. Antibodies to *ZEB1* (1:1000 Proteintech, 21544-1-AP) or b-actin (1:1000 sc-1616) were used. Membranes were washed in TBST, and secondary antibodies (IRDYE 680RD 926-68071 and/or IRDYE 800CW 926-32211, Licor. both 1:5000) were used as appropriate in 1% BSA-TBST solution for 1 h before three washes in TBST and imaged using a Licor Odyssey.

### Preparation of RNA for RNA-seq

RNA samples were diluted to 5 µg using RNAase free water. The first strand cDNA was synthesized using random hexamer primers and M-MuLV Reverse Transcriptase (RNase H-, Promega M5301). Second strand cDNA synthesis was subsequently performed using DNA Polymerase I (Promega, M2051). Double-stranded cDNA was purified using AMPure XP beads (Beckman Coulter, A63881). Remaining overhangs of the purified double-stranded cDNA were converted into blunt ends via exonuclease/polymerase activities. After adenylation of 3’ ends of DNA fragments, NEBNext Adaptor with hairpin loop structure was ligated for hybridisation. cDNA fragments of 150-200 bp in length were generated by PCR and purified using the AMPure XP system (Beckman Coulter, A63881). Transcriptomes were read by Illumina HiSeq platforms by Novogene Inc (Hong Kong, China) in a paired-end manner to a depth of 20 million reads.

#### Bulk RNA-seq bioinformatics analysis

Transcriptomes were read by Illumina HiSeq platforms by Novogene Inc (Hong Kong, China) in a paired-end manner to a depth of 20 M reads. Read quality and adapter content were assessed with FASTQC (v0.11.9)^59^. Reads were trimmed of adapters and quality filtered using cutadapt (v4.4)^60^ to a minimum quality 20 (-q 20) and a minimum read length of 1 (--minimum-length 1) in a paired-end manner. Subsequent alignment of trimmed, paired-end reads was performed using STAR (v2.7.9)^61^ to the reference genome GRCh38 (primary assembly, release 107, https://ftp.ensembl.org/pub/release-107/fasta/homo_sapiens/dna/). Alignment files were output from STAR^61^ in sorted BAM format. Aligned reads were quantified using featureCounts (v2.0.0)^62^. Quantified RNA-seq data were analysed in R (v4.5.1). Count normalisation and differential expression analysis was performed with DESeq2 (v1.48.2)^63^. Normalised counts were regularised log transformed (rlog), and principal component analysis (PCA) was performed on the rlog-transformed matrix using PCAtools^64^ (v2.20.0). Regularised log2 fold change (log2FC) were shrunk using the *lfcShrink()* function of DESeq2^63^ with the *apeglm*^65^ method. Differentially expressed genes were defined using the Benjamini-Hochberg^66^ adjusted *p*-value < 0.05, regardless of log2FC. Gene-level annotation of DEGs was performed using biomaRt (v2.64.0)^67,68^. Over-representation analyses (ORA) were carried out with clusterProfiler (v4.16.0) with org.Hs.eg.db (v3.21.0)^69^ as the annotation database. The gene universe (background) for the ORA was defined as all genes with non-zero DESeq2-normalised counts in the HUVEC samples. For Gene Ontology (GO) analysis, biological process (BP) terms were tested using the *enrichGO()* function. The resulting GO BP terms were simplified using *clusterProfiler::simplify()* (similarity cutoff 0.7, by = “p.adjust”) to reduce redundancy. KEGG pathway enrichment was performed by first converting Ensembl IDs of differently expressed genes (DEGs) to Entrez gene IDs using *bitr()*, then applying *enrichKEGG()* with the same background universe also converted to Entrez IDs. KEGG results were visualised in a readable format (gene symbols) with *setReadable()*. Hallmark gene set enrichment was performed using *msigdbr*^70^ to retrieve *Homo sapiens* gene sets, which were provided to *enricher()* along with DEGs and the same gene universe. From the combined GO, KEGG and Hallmark results inflammation-related and angiogenesis-related terms were selected for visualisation with ggplot2 (v3.4.1)^71^. Heatmaps and hierarchical clustering was generated (Pearson, average linkage) with ComplexHeatmap (v2.24.1) using the rlog-transformed count values. Please see Supplementary data 1 for data.

#### Publicly available scRNA-seq bioinformatics analysis

Single-cell RNA-seq (scRNA-seq) data for choroidal neovascularisation (CNV) endothelial cells (ECs)^18^ were obtained as preprocessed matrices (raw, normalised and batch-corrected counts) from the Murine ECTax database (https://endotheliomics.shinyapps.io/murine_ectax/) along with the metadata and t-SNE coordinates. The raw and processed expression matrices and metadata tables were imported into R (v4.5.1), with downstream analyses using Seurat^72–76^ (v5.3.0). To reproduce the dimensional reduction used in the original publication^18^, t-SNE coordinates from the original dataset were imported and added to the Seurat^72–76^ object with *CreateDimReducObject()*. Endothelial cell (EC) phenotypes were visualised with the *DimPlot_scCustom()* function of the scCustomize (v3.2.0)^77^ package. *Zeb1* expression and density were visualised as a t-SNE plot with *FeaturePlot_scCustom()* and *Plot_Density_Custom()* functions, respectively. Violin plots of *Zeb1* expression were generated with *VlnPlot_scCustom()*. The proportion of *Zeb1* expressing cells was quantified using *Percent_Expressing()* for each phenotype. Differential expression analysis was performed using Seurat’s^72–76^ *FindAllMarkers()* to identify differential expression in each phenotype relative to all others. Please see supplementary data 2 for data.

#### In silico modelling of *ZEB1*

In-silico knockout analysis was performed using scTenifoldKnK^49,78^ (v1.0.2) of lesion EC phenotypes to model the transcriptional consequences of ZEB1 loss. Raw count data for lesion ECs was extracted and filtered to retain genes expressed that have an average expression > 0.1 and excluded ribosomal and mitochondrial genes. The filtered count matrix was used as input to scTenifoldKnK with quality control enabled (*qc* = *TRUE, qc_minLSize* = *0*), specifying Zeb1 to be virtually knocked out (*gKO* = *“Zeb1”*). The scTenifoldKnk pipeline constructs a gene regulatory network from the data, simulates a ZEB1 knockout network by setting the outdegree edges of ZEB1 in the network to zero, and estimates differential regulation (DR) scores between the “wild-type” and virtually perturbed network. Significant differentially regulated genes from the virtual knockout were defined with adjusted *p*-value < 0.05. Functional enrichment of the scTenifoldKnK predicted ZEB1-regulated genes was performed as for bulk RNA-seq, but specifying *Mus musculus* as the organism and using org.Mm.eg.db (v3.21.0)^79^ as the annotation. The average expression of the DR genes was calculated using *AverageExpression()* and the resulting scaled data were used to draw heatmaps with ComplexHeatmap (v2.24.1) for all DR genes, DR genes related to inflammation and angiogenesis derived from the functional enrichment analyses. Please see supplementary data 2 for data.

### Statistics and reproducibility

All statistical analyses were performed using GraphPad Prism (version X). Data are presented as mean ± SEM unless otherwise stated. Statistical tests used for individual experiments are indicated within the corresponding figure legends. Comparisons between two groups were performed using paired or unpaired two-tailed Student’s t tests, as appropriate. Multiple group comparisons were analysed using one-way or two way ANOVA with appropriate post hoc correction. Normality was assessed using the Shapiro–Wilk test where appropriate. A *p*-value < 0.05 was considered statistically significant.

Biological replicates (n) refer to independent experiments performed using separately cultured endothelial cell preparations or independently treated animals. Technical replicates represent repeated measurements obtained within the same experiment. For in vitro assays, experiments were independently repeated at least three times unless otherwise stated. For in vivo CNV experiments, each lesion represented one biological replicate. Sample sizes, statistical tests, and exact *p* values are provided in the figure legends or supplementary datasets.

RNA sequencing, proteomic, and scRNA-seq analyses were performed using established bioinformatic pipelines as described below. Differential expression thresholds and multiple testing corrections are stated within the relevant methods sections. Representative images shown are derived from experiments independently repeated with comparable results. No data were excluded unless predefined technical quality control criteria were not met. Investigators were not blinded during data acquisition unless otherwise stated.

### Reporting summary

Further information on research design is available in the Nature Portfolio Reporting Summary linked to this article.

## Supplementary information

Supplementary Information

Description of Additional Supplementary Materials

Supplementary Data 1

Supplementary Data 2

Supplementary Data 3

Supplementary Data 4

Reporting Summary

## Data Availability

GEO Submissions for ZEB1 knockdown treated HUVECs (GSE333449, GSM9765057, GSM9765058, GSM9765059, GSM9765060, GSM9765061, GSM9765062). Supplementary Data 4 contains the source data underlying graphs. The original Western blot image is included as Supplementary Fig. 1 in the Supplementary Information file.

## Code availability

Code for the analyses presented in this work are publicly visible on GitHub at https://github.com/EndothelialQuiescenceLab/ZEB1_CNV^80^
