# Supplementary material for: Loss of endothelial ZEB1 enhances neovascularisation during choroidal neovascularisation
Source: Commun Biol. 2026 Jul 7;9:1432. doi: 10.1038/s42003-026-10630-0 (PMC13720079; doi:10.1038/s42003-026-10630-0)
Supplement: Supplementary file 2 [file 42003_2026_10630_MOESM1_ESM.docx]

**Supplementary Information**

**Loss of endothelial *ZEB1* enhances neovascularisation during choroidal neovascularisation.**

## Authors

Nicholas Beazley-Long^1,8*^, Joseph L Horder^1*^, Kathryn R Green^1*^, Joshua H Bourne^2^, Amy P Lynch^1^, Nada S Ahmed^1^, Zarah B Tabrizi^1^, Guillermo Diez-Pinel^1^, Christopher P Carroll^3^, Claire L Allen^4^, Charles T Cresswell^1^ Poppy E Harris^1^,Isabela Ferreira^1^ ,Diogo Mosqueira^9^, Julie Rayes^2^, Nigel P Mongan^5^, John King^6^, Chris Denning^9^, Alan McIntyre^3^, Marc P Stemmler^7^, Simone Brabletz^7^, Thomas Brabletz^7^, David O Bates^4^, Andrew V Benest^1^.

**Supplementary Figure 1. Original Westen blot.**

Original Westen blot image corresponding to Figure 6A.
