# Supplementary material for: Loss of endothelial ZEB1 enhances neovascularisation during choroidal neovascularisation
Source: Commun Biol. 2026 Jul 7;9:1432. doi: 10.1038/s42003-026-10630-0 (PMC13720079; doi:10.1038/s42003-026-10630-0)
Supplement: Supplementary file 3 [file 42003_2026_10630_MOESM2_ESM.docx]

**Description of Additional Supplementary Files**

**File name:** Supplementary Data 1

**Description:** Marker analysis between all cell phenotypes; Proportion of cells in phenotypes expressing Zeb1; DE analysis for genes of interest (EndoMT and canonical EC); Proportion of cells in lesion and nonlesion ECs expressing Zeb1; Differential expression analysis between lesion and nonlesion ECs; Differential expression analysis between lesion and nonlesion ECs for genes of interest (EndoMT and canonical EC); In-silico KO of Zeb1 in lesion ECs; Gene Ontology analysis of significant genes identified by in-silico Zeb1 KO in lesion ECs; KEGG analysis of significant genes identified by in-silico Zeb1 KO in lesion ECs; HALLMARKS analysis of significant genes identified by in-silico Zeb1 KO in lesion ECs

**File name:** Supplementary Data 2

**Description:** HUVEC DESeq2 normalised counts; DESeq2 DEG analysis in the direction of ZEB1 KD; Background reference for HUVEC ZEB1 KD DEG functional enrichment analyses; Gene Ontology analysis of DEGs; KEGG analysis of DEGs; HALLMARKS analysis of DEGs

**File name:** Supplementary Data 3

**Description:** cT values for qPCR verification of P-selectin, ICAM and VCAM expression following ZEB1 knockdown in HUVECS.

**File name:** Supplementary Data 4

**Description:** the source data underlying graphs
